# Method for Improving EEG Based Emotion Recognition by Combining It with Synchronized Biometric and Eye Tracking Technologies in a Non-invasive and Low Cost Way

**DOI:** 10.3389/fncom.2016.00085

**Published:** 2016-08-19

**Authors:** Juan-Miguel López-Gil, Jordi Virgili-Gomá, Rosa Gil, Teresa Guilera, Iolanda Batalla, Jorge Soler-González, Roberto García

**Affiliations:** ^1^Department of Computer Languages and Systems, University of the Basque CountryVitoria-Gasteiz, Spain; ^2^Department of Computer Science and Industrial Engineering, Universitat de LleidaLleida, Spain; ^3^Psychiatry Service, Santa Maria University HospitalLleida, Spain; ^4^Biomedical Research Institute of LleidaLleida, Spain; ^5^Department of Medicine, Faculty of Medicine, Universitat de LleidaLleida, Spain; ^6^Institut Català de la Salut IDIAPBarcelona, Spain

**Keywords:** emotions, EEG, eye tracking, biometric information, empathy

## Abstract

Technical advances, particularly the integration of wearable and embedded sensors, facilitate tracking of physiological responses in a less intrusive way. Currently, there are many devices that allow gathering biometric measurements from human beings, such as EEG Headsets or Health Bracelets. The massive data sets generated by tracking of EEG and physiology may be used, among other things, to infer knowledge about human moods and emotions. Apart from direct biometric signal measurement, eye tracking systems are nowadays capable of determining the point of gaze of the users when interacting in ICT environments, which provides an added value research on many different areas, such as psychology or marketing. We present a process in which devices for eye tracking, biometric, and EEG signal measurements are synchronously used for studying both basic and complex emotions. We selected the least intrusive devices for different signal data collection given the study requirements and cost constraints, so users would behave in the most natural way possible. On the one hand, we have been able to determine basic emotions participants were experiencing by means of valence and arousal. On the other hand, a complex emotion such as empathy has also been detected. To validate the usefulness of this approach, a study involving forty-four people has been carried out, where they were exposed to a series of affective stimuli while their EEG activity, biometric signals, and eye position were synchronously recorded to detect self-regulation. The hypothesis of the work was that people who self-regulated would show significantly different results when analyzing their EEG data. Participants were divided into two groups depending on whether Electro Dermal Activity (EDA) data indicated they self-regulated or not. The comparison of the results obtained using different machine learning algorithms for emotion recognition shows that using EEG activity alone as a predictor for self-regulation does not allow properly determining whether a person in self-regulation its emotions while watching affective stimuli. However, adequately combining different data sources in a synchronous way to detect emotions makes it possible to overcome the limitations of single detection methods.

## Introduction

Emotion is a subjective experience characterized by psycho-physiological expressions, biological reactions, and mental states. From a psychological point of view, an emotion is a complex psychological state that involves three distinct components: a subjective experience, a physiological response, and a behavioral or expressive response (Hockenbury and Hockenbury, 2007). Ekman (1999) as part of his valuable legacy, established the foundations for measuring emotions. During his research, he discovered that basic emotions exist and are culturally independent. These basic emotions can be read literally from people's facial expressions. Mehrabian and Russell (1974) proposed that environmental stimuli are linked to behavioral responses by the primary emotional responses of arousal, pleasure, and dominance. Russell and Pratt (1980) presented his two-dimensional model of affect, including pleasure-displeasure and arousal-sleep as the two dimensions. The model itself is based on the internal representation of affect of the people tested, i.e., their cognitive structure of the interpretation of emotions and not a description of their current affective state. The result is a universal and easily transferable metric for describing emotions. Dimension-based approaches are widely used in affective computing for measuring emotions (Calvo and D'Mello, 2010). According to Picard (1997), most theories about emotions can be divided in two main groups. The first group states that emotions are cognitive, emphasizing their mental component, while the second group states that emotions are physical, thus emphasizing their corporal component. However, some emotions are not entirely corporal or have a pure emotional component, they may also have a strictly cognitive part associated. An example is empathy, in which two different emotional and cognitive facets can be determined (Davis et al., 1987).

Regarding systems that can help studying the affective processes in the human brain, EEG, PET scans, or fMRI stand out. EEG can detect changes in brain activity over milliseconds, which is excellent considering an action potential takes ~0.5–130 ms to propagate across a single neuron, depending on the type of neuron. Nevertheless, EEG measures the brain's electrical activity directly, while fMRI and PET record changes in blood flow or metabolic activity, which are indirect markers of brain electrical activity. EEG can be considered as the most studied potentially non-invasive brain activity detector system in computational emotional systems, mainly due to its fine temporal resolution, ease of use or portability, been not as invasive as the other alternatives and relatively low cost. However, EEG alone does not record indirect brain electrical activity markers, such as changes in blood flow or metabolic activity. Therefore, combining EEG with other non-invasive biometric information detection systems is a promising way to improve emotion detection.

Devices for biometric measurement are nowadays widely available. Data from wearable sensors can be used for passive tracking of physiological responses (Morris and Aguilera, 2012). The range of available devices is very large, including wristbands, accelerometers, posture sensors, or cameras, which serve to measure biometric data such as temperature, pulse or skin conductance.

Regarding other interesting biometric signals usable for emotion detection, ElectroDermal Activity (EDA) is the property of the human body that causes continuous variation in the electrical characteristics of the skin. These changes in the electrical properties of the skin are caused by an interaction between environmental events and the psychological state of the individual. Human skin is a good conductor of electricity and when a weak electrical current is induced on the skin, changes in the skin conductivity can be measured. Öhman et al. (1993) discussed the role of autonomic activity in emotion, focusing on EDA as a reliable estimator. On the other hand, Appelhans and Luecken (2006) provided a review of the theoretical and empirical rationale for the use of Heart Rate Variation as an index of individual differences in regulated emotional responding.

Another interesting source of biometric measure about emotion is human vision, which transmits the images it receives via the eye, retina, and second cranial nerve (optic nerve) into the brain passing the chiasm, thalamus, and visual cortex. Moreover, it tries to control both eyes, directing them to the most relevant position at that point in time. This task is performed by the oculomotor system that controls the six muscles attached to each eye via the third cranial nerves. Eye tracking provides human vision feedback. The data collected by the eye tracker captures the moment when the image is shown followed by the response of the oculomotor system, including response time and exact saccade characteristics. Data regarding blink frequency and pupil size, which can indicate cognitive load changes, can also be accessed by these means. Besides, the most desired type of eye tracking output is the estimation of the Point Of Regard (POR) of the viewer, that is, where he/she is looking. By detecting eye position, gaze direction, sequence of eye movement and visual adaptation during cognitive activities, eye tracking is an effective tool for experimental psychology and neurological research, as it can allows establishing which aspects of EEG or biometric signals may be related to human vision (Popa et al., 2015).

In fact, vision becomes a clue for understanding emotions and becomes a first step to understand what is happening emotionally. We must consider that physical activity not detectable by EEG alone has influence on the brain activity. Unintentional body movements have their influence in the EEG. Eye movements introduce large artifacts to EEG and thus render data analysis difficult or even impossible. Trials contaminated by eye movement and blink artifacts have to be discarded, hence in standard EEG-paradigms subjects are required to fixate on the screen. Plöchl et al. (2012) aims at a better understanding of individual eye movement artifacts, their interrelations and the respective implications for eye artifact correction. Apart from eye tracking, other biometric signals such as blood variations and heart rate have also been used to more appropriately interpret brain signals. For instance, De Pascalis et al. (1998) recorded EEG and heart rate during self-generated happy and sad emotions using a relaxation condition as a control after the administration of a standard hypnotic induction. Such biometric signals have to be considered in order to isolate and later discard their associated brain activity from the emotion analysis. In order to differentiate the neural substrates of vagal tone due to emotion, Lane et al. (2009) correlated HF-HRV with measures of regional cerebral blood flow derived from PET and 15 O-water in 12 healthy women during different emotional states.

Self-regulation with different bio signals viewing affective imagery was analyzed by Davidson and Schwartz (1976), who performed an experiment to obtain information on central mechanisms underlying cardiac self-regulation by comparing changes in cerebral asymmetry during self-control of heart rate with changes observed during the production of affective imagery. Heart rate showed significant effects between up vs. down, and between anger vs. relaxing imagery in the image phase. The EEG data indicated similar patterns of hemispheric asymmetry.

We have performed a study to determine the validity of proposed process in which it is aimed to determine whether EEG activity alone is a good indicator of self-regulation when viewing affective images. A group of forty-four people had to watch a series of affective stimuli while their EEG activity, biometric signals, and eye position were synchronously recorded. The EDA data were processed according to existing literature to determine which people self-regulated their biometric activity while watching affective stimuli. Participants were divided into two groups, the ones that self-regulated and the ones that did not according to EDA.

The hypothesis of the work was that people who self-regulated would show significantly different results when analyzing their EEG data. In order to prove it, different machine learning algorithms for emotion recognition were used for both groups and their results compared.

Our results show that using EEG activity alone as a predictor for self-regulation does not allow properly determining whether a person in self-regulation its emotions while watching affective stimuli. This fact shows that adequately combining different data sources in a synchronous way to detect emotions allows overcoming possible deficits when using single detection methods. The use of un-invasive devices for different signal detection will be favored in order to maintain user interaction as natural as possible.

To validate this hypothesis and provide information about how the process performs, the next sections present the study in which eye tracking, biometric, and EEG signals have been combined for determining basic and complex emotions. Gathered information has been used to detect different emotions the participants experienced while watching a predefined set of stimuli.

## Materials and methods

### Ethics statement

The participants were informed about the possible discomforts deriving from the experimental protocol and agreed to participate through an informed consent. The study was carried out according to the principles of the declaration of Helsinki and approved by the ethics committee on clinical research of the Arnau de Vilanova University Hospital.

### Participants

Forty-four participants (6 males and 38 females, aged between 20 and 30 years with a mean of 22 years) participated in the study. The participants were undergraduate students of the University de Lleida. Individuals with a prior history of neurological or psychiatric illness were excluded.

### Emotion-eliciting images and videos

Thirty-one images were retrieved from the International Affective Picture System (IAPS) (Lang et al., 1999) to elicit positive (Eleven pleasant images), negative (Thirteen unpleasant images) or neutral (7 neutral images) emotions. Images were presented at intervals of 5 s and neutral images were placed after the images producing the higher values of valence to test if participants did return to a neutral state or they just reduced their excitement slightly. IAPS is a database of images designed to provide a standardized set of pictures for studying emotion and attention widely used in psychology, and emotion research (Horlings et al., 2008; Petersen et al., 2011). Each picture is accompanied by a detailed list of average ratings of the emotions elicited, accounted for by valence, arousal, and dominance dimensions. Apart from the images, four video clips of 15 s each were also showed to the participants, illustrating positive and negative emotions. These videos were taken from Mind Reading Full Version 1.2 software and were displayed after the images. To avoid order effects on emotion elicitation, the presentation of the 31 images was counterbalanced using a Latin square design.

### EEG, biometric signal, and eye tracking measurement

EEG was recorded using Emotiv EPOC EEG. It has a headset with sensor to capture 14 EEG channels plus 2 references. Channel names based on the international 10–20 electrode location system are: AF3, F7, F3, FC5, T7, P7, O1, O2, P8, T8, FC6, F4, F8, AF4, with CMS/DRL references in the P3/P4 locations. Emotiv EPOC/EPOC + uses sequential sampling method, single ADC, at a rate of 128 SPS or 256 SPS (2048 Hz internal). Emotiv EPOC operates at a resolution of 14 bits or 16 bit per channel with frequency response between 0.16 and 43 Hz.

The technology used in the experiment also included an Empatica Wristband. This bracelet has sensors to capture different biometric signals at the same time, specifically EDA, Blood Volume Pulse (BVP), Acceleration, Heart Rate, and Temperature.

**Figure 1:**
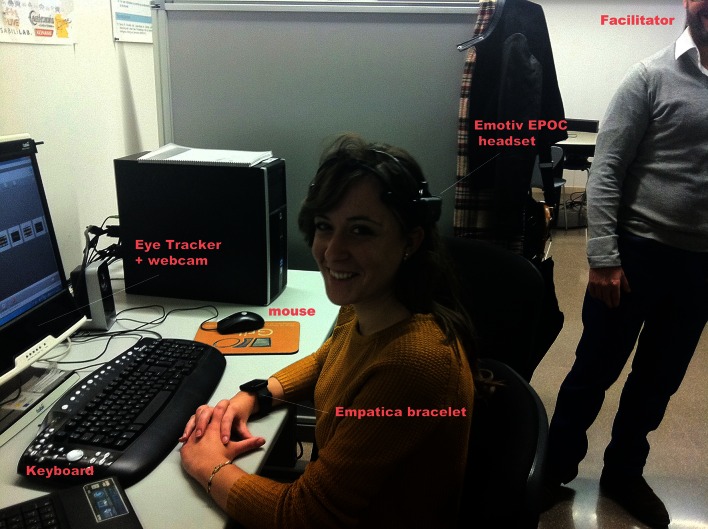
**Participant during the study using the Tobii T60 Eye Tracker, Empatica wristband, and Emotiv EPOC EEG headset**. Authors have the approval of the depicted woman to have her photo published.

Tobii T60 Eye Tracker was used for eye tracking, which has infrared diodes to generate reflection patterns on the corneas of the user's eyes. These reflection patterns, together with other visual information about the person, are collected by image sensors and enable calculating the POR in real time. Figure 1 shows a participant wearing EEG and biometric signal capture devices while using the eye tracker.

The eye tracker is based on the pupil center corneal reflection (PCCR) technique (Duchowski, 2007). The user eye is illuminated with near-infrared light and the reflections are captured by the two eye tracker cameras. Both images are then processed to identify the reflection of the light source on the cornea (glint) and in the pupil. This information is used to calculate the vector formed by the angle between the cornea and pupil reflections, which allow computing gaze direction and point relative to the image shown to the user in the eye tracker monitor. This stream of data is processed by Tobii Studio software to compute gaze plots, which show the location, order, and time spent looking at locations on the stimulus. It is also possible to use Tobii Studio to generate heat-maps that show how looking is distributed over the stimulus. Both gaze plots and heat-maps are then used in data pre-processing, as described in Subsection Data Pre-processing.

### Hypothesis

The main hypothesis of this work is summarized next:

H1–Self-regulation affects emotions recognition in EEG signals.

People whose self-regulated during the session was determined by means of EDA would show significantly different results when analyzing their EEG data with different machine learning algorithms for emotion recognition.

### Experimental protocol

The test was carried out in a usability laboratory located at the Universitat de Lleida. Before the execution of the tasks defined in the protocol, the facilitator provided the participants with the pre-test questionnaire they had filled in, in which their demographics and their suitability for the study were established. If the participant was suitable for the study, the experimental procedure started after all devices were checked and calibrated in order to synchronize them and data collection began. The facilitator explained the procedure to the participants and helped them to wear the devices and check that they were well placed and working correctly. The facilitator drove the experimental protocol and assisted them during the requested tasks. The facilitator also took care that anybody or anything disturbed the experiment. The requested tasks were basically to observe the set of 31 IAPS pictures and 4 videos from the MindReading corpus. Each image was shown to each user for 5 s. To avoid order effects on emotion elicitation, which could negatively bias the execution of the tasks, the presentation of the thirty-one images was counterbalanced using a Latin square design. When the participants finished all tasks, which took about 15 min overall, the facilitator asked them some post-test questions and briefly interviewed them to detect issues experienced by the participants during the test.

### Data managing

All used devices were synchronized and used jointly during the experiments. Regarding data analysis, the following phases have been performed.

#### Data pre-processing

Input from different devices is combined to help filtering out undesired signals. First, heat-maps and gaze plots are obtained using the eye tracker and pre-processed. The eye tracker helps deciding which part of the combined set of signals produced by the different sensors used in the experiment is relevant. It shows what the user is visualizing and helps correlating this with the measures of the bracelet and the headset, thus making it possible to relate what the user is looking at and what his/her feelings are. Moreover, this way it is easier to disregard undesirable effects in the EEG, such as blinking or head or muscle movements, as they are recorded by the Eye Tracker.

Another combined signal is the accelerometer, which helps discarding non-relevant signals coming from voluntary or involuntary user movements. These undesirable signals can be removed when detected using software filters. These filter have been implemented with EEGLab, an interactive Matlab toolbox for processing continuous and event-related EEG. In order to analyze EEG data, several filters have been applied using Matlab and EEGLab, specifically a passband filter (0.1–50 Hz), and a notch filter (50 Hz) to avoid parasite power signal.

Besides, Independent Component Analysis (ICA; Karhunen et al., 1997) was performed on filtered data removing artifacts, such as eye blinks, which were detected by eye tracking, from EEG data. ICA was run once, then bad epochs were rejected, ICA was ran another time on pruned data and the resulting ICA weights were applied to the same dataset. The disadvantage of ICA is that the components do not necessarily only contain artifact data, they might also contain underlying EEG data. Thus, removing the contaminated component will lead to the loss of EEG data. Consequenty, more sophisticated approaches that improve the ICA algorithm have been explored (Plöchl et al., 2012; Mannan et al., 2016). Particularly, methods that combine the ICA algorithm and wavelets (Burger and van den Heever, 2015), which seems to be a promising approach.

#### Data processing

The segments of EEG data selected during pre-processing (called Epochs) are processed using eye tracker guidance, based on heat-maps and gaze plots. EEG data lacks time stamps, so synchronization with Eye Tracker data, which is time stamped, is attained using a loud sound. The sound is recorded together with Eye Tracker audio and appears in the EEG signal due to the user response to the sound.

Combining EEG and Eye Tracker data allows knowing the time expended at different parts of the user interface (in our case the pictures from the IAPS set shown to user), and even the order they have been visualized. As during pre-processing, this analysis is also performed using EEGLab, so it is possible to filter the EEG Epochs that correspond to the time periods while the user was looking at a particular IAPS picture or even an area of it.

Then, EDA is also included in the analysis. In this case all Empatica Wristband data is time stamped and thus synchronization is eased. This additional information will be considered later during data analysis based on the fact that changes in EDA occur as a result of activation of regions deep in the brain. This is sustained by previous work showing that when the amygdala or hippocampus get activated, they elicit skin conductance on the same side of the body (Mangina and Beuzeron-Mangina, 1996).

For instance, stimulating four brain regions on the left or four on the right (with depth electrodes) produces large skin conductance on the same side. Finally, we obtain valence from EEG data analysis and arousal values from EDA analysis. High/low arousal values are candidates to choose the same temporal EEG values.

#### Data analysis

After data processing, all the signals to be considered during the analysis are already filtered and synchronized. Data is then combined by organizing it on the basis of the provided stimuli, i.e., the IAPS images. For each stimulus, all the EEG and EDA measures in the corresponding time range are processed to compute valence and arousal.

EEG is used to compute the valence based on frontal asymmetry, as previously introduced and detailed in Section Data Analysis Remarks. On the other hand, EDA is directly used as a measure of the arousal. The valence and arousal values combined define a point in the Pleasure Arousal and Dominance (PAD) 2D space (Mehrabian and Russell, 1974). This point corresponds to the emotion, which is then correlated with the valence and arousal values reported in IAPS for each of its images.

### Data analysis remarks

Some preliminary analysis has been performed over empathy complex emotion. It has been found significantly higher P300 amplitudes on parietal electrodes (Kleih and Kübler, 2013) in participants with a low ability for perspective taking and therefore, lower empathy, as compared to participants with higher empathy. As empathy can be divided in cognitive and emotional facets (Davis et al., 1987) and P300 belongs to its cognitive facet, the analysis is performed strictly over the emotional facet. Empathic Gaze pattern was examined where expressions of sadness and anger attracted more gaze dwell-time to the eye-region than other emotional expressions (Cowan, 2015). Calvo and Lang (2004) began to work in gaze patterns when looking at emotional pictures and there has been intensive work in measuring emotions with eye tracking (Raudonis et al., 2013). These works have been taken into account when analyzing gathered data.

Following Tóth (2015), it is possible to establish positive vs. negative emotions using EEG analysis, as positive emotions such as joy, amusement or excitement are mostly processes in the left hemisphere of the brain, while negative ones, such as disgust, fear, sadness are handled in the amygdala and the right hemisphere, which gives rise to the phenomena of frontal alpha asymmetry. Self-referential emotions such as pride, shame or guilt can also be found, as they operate in the frontal lobe and arise when our behavior is compared to social norms. Arousal is measured from skin conductivity, which is driven by the sympathetic branch of the autonomic nervous system and is used to capture the affective state. Galvanic Skin Response (GSR) can be divided into two main features: Skin Conductance Level (SCL), the tonic part, provides a measure of arousal while Skin Conductance Response (SCR), the phasic part, reflects instant changes in arousal. SCR present quick changes in excitement, and corresponds to fast changes in sympathetic arousal, and represent general vigilance. Table 1 is used for describing the relationships between different emotions and their biometric correlates.

**Table 1:** **Emotions and their biometric correlates according to Tóth (2015)**.

**Emotions**	**GSR**	**BVP**	**Temperature**
Anger	Decreases	Increases	Increases
Fear	Increases	Increases	Decreases
Happiness	No change	Normal	No change

### Machine learning experiments

Derived from the gathered EDA data, the participants were divided into two groups, one group in which participants self-regulated when watching the affective stimuli and another group for the users that did not self-regulate. From the forty-four participants, the data of one of them was discarded, as it was not synchronized appropriately due to a hardware malfunction. Out of the other 43, EDA activity showed that 21 self-regulated and were assigned to group one, while the other 22 were assigned to group number two.

The experiments were carried out using 10 well-known machine-learning supervised classification algorithms through the Weka software package (Hall et al., 2009), which includes a collection of machine learning algorithms for data mining tasks. A brief description of the classifiers is presented below.

1. Support Vector Machines (SVM): Set of related supervised learning methods used for classification and regression (Cortes and Vapnik, 1995). Viewing input data as two sets of vectors in a *n*-dimensional space, an SVM will construct a separating hyperplane in that space, one that maximizes the margin between the two datasets.
2. Logistic (L): Class for building and using a multinomial logistic regression model with a ridge estimator (Le Cessie and van Houwelingen, 1992).
3. Random Forest (RF): Constructs a combination of many unpruned decision trees (Breiman, 2001). The output class is the mode of the classes output by individual trees.
4. Repeated Incremental Pruning to Produce Error Reduction (RIPPER): Rule-based learner presented in Cohen (2005) that forms rules through a process of repeated growing (to fit training data) and pruning (to avoid overfitting). RIPPER handles multiple classes by ordering them from least to most prevalent and then treating each in order as a distinct two-class problem.
5. k-Nearest Neighbors (KNN): This algorithm is a case-based, nearest-neighbor classifier (Aha et al., 1991). To classify a new test sample, a simple distance measure is used to find the training instance closest to the given test instance, and then, it predicts the same class as this nearest training instance.
6. Naive Bayes (NB): The naive Bayes rule uses the Bayes theorem to predict the class for each case, assuming that the predictive genes are independent given the category (Cestnik, 1990).
7. Logistic Regression (LR): A logistic regression is considered as a regression model where the dependent variable is categorical (Freedman, 2009).
8. C4.5: It represents a classification model by a decision tree (Quinlan, 1993). The tree is constructed in a top-down way, dividing the training set and beginning with the selection of the best variable in the root of the tree.
9. Radial Basis Function (RBF) network: A radial basis function network is an artificial neural network using radial basis functions as activation functions (Broomhead and Lowe, 1988). The output of the network is a linear combination of radial basis functions of the input neuron parameters.
10. Multilayer Perceptron (MLP): A multilayer perceptron is a feedforward artificial neural network model to map sets of input data onto a set of appropriate outputs (Rosenblatt, 1961). An MLP consists of multiple layers of nodes in a directed graph, with each layer fully connected to the next one. Except for the input nodes, each node is a processing element with a nonlinear activation function.

Selected hyperparameters and configuration options for each classifier were: C 1.0 and Polykernel with a 1.0 value for its exponent (SVM); 1.0E-8 for ridge estimator (L), unlimited tree depth and 100 iterations to be performed (RF); 3.0 as the minimum total weight of instances in a rule and 3 optimization runs and 10-folds for pruning (RIPPER); 1000 neighbors and linear nearest neighbor search (KNN); crossvalidated with heuristic for greedy stopping and 500 maximum iterations for logicboost (LR); 0.1 as confidence factor for pruning and at least 15 instances per leaf (C4.5); 2 clusters and 0.01 as minimum standard deviation for the clusters (RBF); and 1000 epochs to train through, (attribute + instance)/2 hidden layers on the neural network, 0.4 learning rate and 0.3 momentum (MLP).

In all of the experiments, 10-fold cross-validation (Stone, 1974) was applied to get validated classification accuracy (well-classified rate), which is the main measure that has been used in this study to evaluate classification methods. The accuracy reflects how many times the emotions are recognized. In this case, three categories of emotions were defined: pleasant, unpleasant, or neutral, according to the IAPS images that participants viewed. Accuracy is expressed as a percentage with respect to the total of the recordings.

## Results

In this section, different kinds of results are provided. First, the outcome of the machine learning experiments is presented, as they serve to evaluate proposed hypothesis. Next, it is described how basic emotions are determined regarding their valence and arousal in the 2D PAD space and, then, the results for the complex emotion empathy.

### Valence and arousal for basic emotions

**Figure 2:**
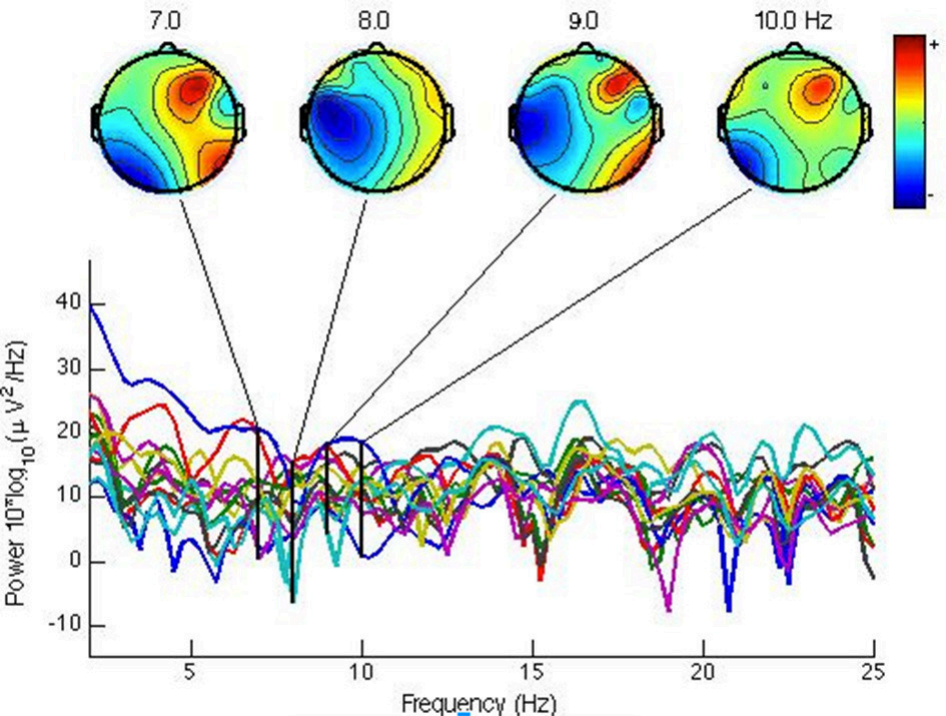
**Brain activity spectrum for the alpha band (7 to 10 Hz) showing in all cases more activity in the right hemisphere, indicating unpleasant valence for a mutilation picture (IAPS Database)**. The bottom part shows the values for each individual headset sensor across all bands, linked to the 4 aggregated views of the overall brain activity at the 7, 8, 9, and 10 Hz bands shown at the top.

**Figure 3:**
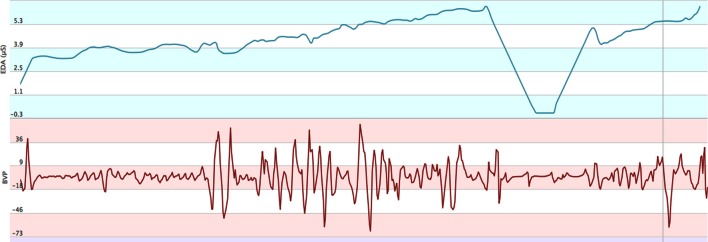
**Screenshot of Empatica web interface showing two of the measures being monitored, ElectroDermal Activity (EDA), and Blood Volumetric Pressure (BVP)**.

With the data collected by Emotiv Headset, it can be observed that the right hemisphere was clearly generating more alpha waves between 7 and 10 Hz (see Figure 2) when users saw unpleasant images. This phenomenon is called frontal asymmetry and introduced in previous sections. In fact, this example corresponds to an IAPS image with 1.59 valence and 7.60 arousal. It has been cataloged as unpleasant. Thus, it is possible to establish a dichotomy between pleasant/unpleasant, called valence. At the same time, all these results can be related to GSR and BVP measures too, captured by the Empatica device (see Figure 3). A correlation, according to the work by Tóth (2015), can be established. Accelerometer measures were used to discard signal artifacts due to muscles activity, whereas HB (Heart Beat) was used as a measure of excitement and validated in the post-test to rule out users that started the test in an over-excited state that negatively affected the experimentation session.

### Complex emotions: empathy

**Figure 4:**
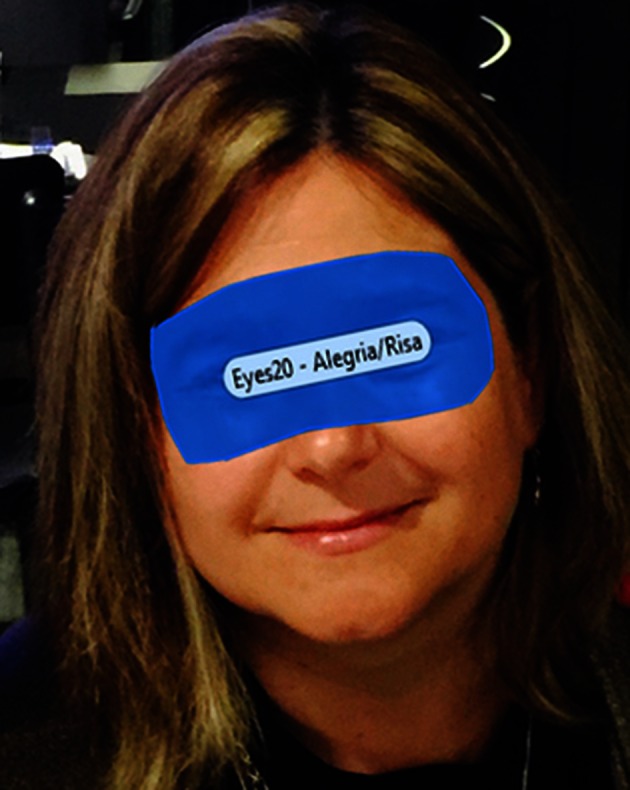
**Eyes zone definition technique over pictures**.

As it is mentioned in the Introduction, when dealing with more complex emotions, such as empathy, we must distinguish two facets, the cognitive and the emotional one. In this work we have focused on the emotional facet, the most complex to measure. Following Cowan (2015) gaze empathy patterns, bounded regions were drawn using Tobii Studio around eyes (see Figure 4) and mouth. The percentage of gaze time fixation on eyes (see Figure 4) and mouth were computed then separately by the eye tracker, with the intention of discerning empathic people. The basis for this classification is that empathy is correlated positively to the percentage of gaze time in the eyes area. This approach was validated by Wang et al. (2006), who used this technique with empathic tutoring software agents, as a way to measure empathy without getting deeper into distinguishing between cognitive and affective empathy. This part of the analysis was performed over the videos alone.

**Figure 5:**
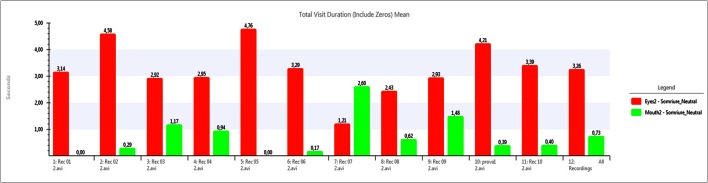
**Eye fixation times (in seconds) compared to Mouth fixations (in seconds) for a neutral video**. Measures for each individual user (1 to 11) and average (12).

**Figure 6:**
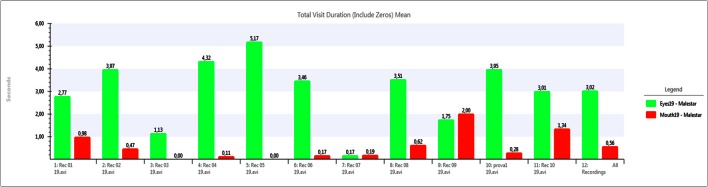
**Eye fixation times (in seconds) compared to Mouth fixations (in seconds) for an unpleasant video**. Measures for each individual user (1 to 11) and average (12).

**Figure 7:**
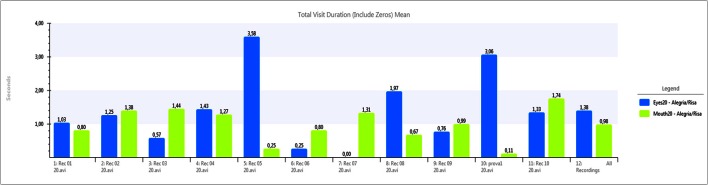
**Eye fixation times (in seconds) compared to Mouth fixations (in seconds) for a pleasant video**. Measures for each individual user (1 to 11) and average (12).

Using the data collected by the eye tracker, Figures 5–7 show a comparative between time fixations on eyes and mouth. In both males and females, the percentage of gaze eye time fixation is always bigger than the percentage of gaze mouth time fixation. In just one case, a female never looked at another women' eyes, just to men's eyes alone and just a few times; she preferred to occasionally look at the mouth. She presented a small degree of deafness. Another extreme case was found in another woman, who focused on eyes almost the whole time (over 90%). In this case, rather than being empathetic, the person was found to be inquisitive, corroborated by the fact that her EDA activity was flat.

**Figure 8:**
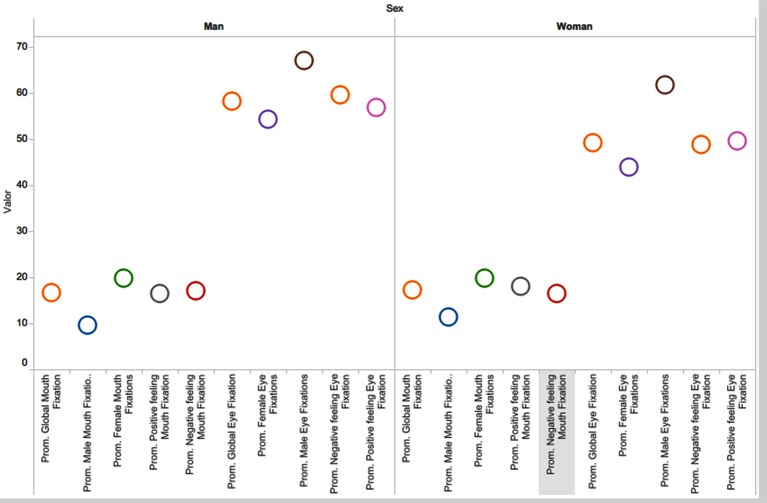
**Male/female gaze patterns (in each case values are percentages, Global Mouth fixations, Male/Female Mouth fixations, Positive/Negative feeling Mouth Fixation, and the same for Eye Fixations)**.

We also analyzed the differences between females and males, although the amount of men was small compared to women. There were not substantial differences in the percentage of mouth fixations, as it can be seen in Figure 8. Only in the percentage of eye gaze fixations there is a small difference value between males as females, being bigger for men than for women.

In Table 2, the relationship between the percentage of gaze eye fixations and EDA values is shown. Antidepressants alter EDA values, mostly the phasic activity. This information was collected in the pre-test and correlated to EDA values where tonic goes down. We related participants with EDA self-control to values above 50% gaze eye fixations. The exception was people consuming antidepressants, concretely 3 participants.

**Table 2:** **Phasic and tonic EDA related to gaze time percentage activity**.

**EDA**	
0.01	56.27
0.04	65.34
0.05	81.49
–	39.5
Flat	50.67
Great phasic peaks	57.74
Little phasic peaks	36.07
Moderated phasic activity	38.85
Tonic goes down, little phasic activity	56.94
Tonic goes down, no phasic activity	47.86

*Percentages above 50 are marked in green, higher values are highlighted with darker green*.

Table 3 summarizes the descriptive statistics for the numerical measures collected during the study. These numerical measures include the percentages of fixations in the eye tracking and the minimum and maximum blood pressure values. The table displays obtained overall values and also shows them per female and male.

**Table 3:** **Descriptive statistics for the numerical measures collected during the study**.

	**Count**	**Mean**	**Var**.	***St. Dev***.	**Min**	**Max**
**OVERALL**						
Global eye fixation	44	50,468	372,720	19,306	1,542	86,204
Positive feeling eye fixation	44	50,632	388,632	19,714	2,261	91,106
Negative feeling eye fixation	44	50,305	380,752	19,513	0,823	83,256
Male eye fixations	44	62,416	385,179	19,626	1,110	92,186
Female eye fixations	44	45,348	407,737	20,193	1,727	86,102
Global mouth fixation	44	17,159	175,209	13,237	0,000	52,453
Positive feeling mouth fixation	44	17,770	175,027	13,230	0,000	53,060
Negative feeling mouth fixation	44	16,547	189,950	13,782	0,000	57,969
Male mouth fixations	44	11,105	117,774	10,852	0,000	53,560
Female mouth fixations	44	19,753	219,041	14,800	0,000	59,645
BPM min	41	73,829	186,695	13,664	47,000	102,000
BPM max	41	91,098	315,040	17,749	55,000	137,000
**WOMEN**						
Global eye fixation	38	49,249	381,522	19,533	1,542	86,204
Positive feeling eye fixation	38	49,660	392,246	19,805	2,261	91,106
Negative feeling eye fixation	38	48,838	393,598	19,839	0,823	83,256
Male eye fixations	38	61,685	411,062	20,275	1,110	92,186
Female eye fixations	38	43,919	412,282	20,305	1,727	86,102
Global mouth fixation	38	17,225	167,431	12,940	2,394	52,453
Positive feeling mouth fixation	38	17,980	161,275	12,699	2,379	53,060
Negative feeling mouth fixation	38	16,469	187,681	13,700	1,669	57,969
Male mouth fixations	38	11,338	124,748	11,169	0,000	53,560
Female mouth fixations	38	19,748	203,489	14,265	1,812	59,645
BPM min	35	72,914	174,081	13,194	47,000	99,000
BPM max	35	88,886	283,163	16,827	55,000	120,000
**MEN**						
Global eye fixation	6	58,192	299,246	17,299	32,170	83,150
Positive feeling eye fixation	6	56,789	386,943	19,671	26,207	84,453
Negative feeling eye fixation	6	59,595	241,927	15,554	38,133	81,847
Male eye fixations	6	67,050	240,846	15,519	49,405	89,716
Female eye fixations	6	54,396	341,909	18,491	24,783	80,336
Global mouth fixation	6	16,739	267,564	16,357	0,000	45,809
Positive feeling mouth fixation	6	16,442	309,349	17,588	0,000	49,908
Negative feeling mouth fixation	6	17,037	244,400	15,633	0,000	41,710
Male mouth fixations	6	9,631	86,700	9,311	0,000	24,840
Female mouth fixations	6	19,786	377,933	19,440	0,000	54,796
BPM min	6	79,167	269,767	16,425	64,000	102,000
BPM Max	6	104,000	360,800	18,995	84,000	137,000

### Machine learning experiments

Table 4 shows the results obtained for each group when a classification algorithm is applied for emotion recognition, in addition to the mean values and standard deviation (SD) for each group of users. The best accuracies obtained per group of participants are highlighted in bold. The results suggest that there is no clear algorithm that performs better. L is the algorithm that performs best for Group1-Non self-regulated (40.9091%), while MLP shows the best results for Group2-Self-regulated (42.7035%), and Ripper (42.4606%) for all users together. There are also no relevant differences among classifiers inside each group, with the exception of G2, in which NB shows poor results compared to the rest (29.8003% compared to a mean of 37.9416%), and MLP betters by more than a 1% the outcome of the second best algorithm.

**Table 4:** **Accuracy percentages for each group of study participants using classifiers**.

**Algorithm**	**G1-Non-self-regulated**	**G2-Self-regulated**	**All**
SVM	36.8035	38.7097	39.0098
L	35.1906	37.9416	34.5086
RF	40.0293	39.4777	41.2603
RIPPER	39.8827	39.9386	**42.4606**
KNN	35.4839	37.3272	35.9340
NB	34.3109	29.8003	32.2581
LR	**40.9091**	41.1674	42.0855
C4.5	35.1906	33.9478	33.5334
RBF	35.7771	38.4025	39.6099
MLP	40.6158	**42.7035**	42.1605
Mean	37.4194	37.9416	38.2821
SD	2.61820374	3.694443085	3.897600106

*Best algorithm for each group marked in bold*.

Following García et al. (2010), we employed two non-parametric methods to compare multiple algorithms, the classical Friedman test (Friedman, 1937), and a modification by Iman and Davenport (1980) to detect statistical differences among the different classification paradigms. In this case, none of the tests rejected the null hypothesis of equivalence between groups of participants, since the *p*-values (0.2725 and 0.2855 respectively) are not lower than the α-value (0.05). In order to complement these results, Friedman's Aligned Rank Test and Quade Test presented in García et al. (2010) were also used and also did not reject the null hypothesis, as their *p*-values were 0.3603 and 0.4396 respectively. Consequently, the resulting *p*-values indicate that we cannot reject the null hypothesis that all the algorithms perform the same, we did not proceed with a *post-hoc* test.

## Discussion

The goal of this study is to establish the validity and the limitations of a methodology for using devices for eye tracking, EEG and biometric signal measurement for the study of emotion in ICT environments. As the objective was that users should behave as naturally as possible in the ICT environment, the devices used for different signal data collection were selected to be the least invasive possible. The results show that it is possible to adequately combine different data sources to detect both basic and complex emotions with devices capable of being used in ICT environments.

Regarding basic emotions detection, there are several models of emotions that can be used to determine a given emotion. After reviewing the literature, the model defined in the Pleasure Arousal and Dominance (PAD) 2D space (Mehrabian and Russell, 1974) was used to characterize biometric measures. EEG and EDA are used to get the Valence (Pleasure) and Arousal values that determine the emotion in the PAD 2D space. On the other hand, for complex emotions, empathy has been detected using eye tracking and biometric signals, mainly EDA.

### Invasiveness when gathering biometric information

Using EEG with other biometric devices requires an appropriate synchronization among them in order to be used in real ICT environments where the requirements are that they must work in real-time and not be invasive. In this regard, invasiveness in HCI environments is not understood exactly as in other areas in which EEG is used, such as in medicine. The presence of any annoying object is enough to be considered invasive in HCI, as users would not be able to interact with the ICT environment in a natural way. Therefore, the devices for EEG data gathering in HCI tend to be less invasive and, as a consequence, include less EEG channels than the devices used in medical environments. As a consequence, the data gathered is not as accurate in HCI environments as it is in medicine. This sacrifice is necessary in order to preserve user comfort.

Fortunately, such loss of accuracy does not imply that emotions recognition cannot be still performed accurately with the devices for EEG available in HCI. Emotiv EPOC EEG headset used in this work includes sensors to capture 14 EEG channels plus 2 references, which has proven to be enough to measure valence. This measure is combined with the arousal, determined by means of a non-invasive wristband, to determine user emotion using a PAD model based on the 2D space defined by the valence and arousal dimensions. This hardware for EEG detection has also been used in other studies for emotion recognition based on EEG, such as Sourina and Liu (2011).

In fact, the most recent prototypes of EEG devices are becoming even less invasive, at the risk of losing even more recall when acquiring EEG data. They are shaped like tiaras and their electrodes do not need to be hydrated, such as in the case of the Muse device^1^ With respect to emotion recognition, such devices can record enough information to determine frontal alpha asymmetry related to negative emotions, as the emphasis is on the difference between hemispheres. Besides, software algorithms performed over different measures improve accuracy with the use of machine learning techniques. The economic aspect is also a limitation in this regard, although the aim of such devices is not to detect diseases, which requires greater precision in the technology used.

### Benefits of signal combination

As it has been mentioned throughout the article, the combined use of different devices to gather different kinds of signals, like eye tracking, EEG or biometric signals has been useful to determine emotions. Regarding the advantages of the combined use, there are several aspects that are worth being taken into account as detailed below.

Both pre-tests and post-tests have been crucial to detect relevant aspects associated with the study, going from drug consumption (antidepressants) to deafness detection. Both tools are relevant to contextualize biometric data gathered in the study.

The eye tracker device has proved to be a useful tool in order to visualize what the user is really doing and a useful complement of other sources. Therefore, its use is highly advocated whenever possible, although its economic cost is also an aspect to be taken into account. As it has been explained in the previous sections, it can be used to detect empathy combined with EDA. Besides, more patterns of gazing explorations were found. Although they have not been related to emotions yet, some users did not look at the context of the image, they just focused on the problem or disease displayed in the images. This is work in progress and we are currently exploring the correlations with other variables recorded with the different devices.

EDA activity has also proven very useful. It is divided into phasic and tonic. The tonic part provides a measure of arousal and is related to mood, while the phasic part, which reflects instant changes in arousal, represents quick changes in excitement and is related to individual stimuli. For this study, it has been important to have devices that allowed us to measure and monitor both of them. Additionally, the values gathered by the accelerometer, heartbeats and BVP have been used to discard or to evidence some other problems as movement or excitement about something.

Altogether, we have shown that biometric measures that detect emotional responses can be used to correlate to information coming from questionnaires or to detect consistencies and inconsistences between gathered information and self-reports or observations gathered during tests. Interviews and close observations are important to determine the reasons for various biometric measures, such as the nervousness of the participants.

As the combination of devices makes it feasible to implement different preprocessing and data analyzing techniques, using software other than that used for this study may be important for a more accurate analysis. In this sense, BCILab software is being evaluated for use in subsequent experiments.

More biometric information gathering methods can be added. For instance, Rainville et al. (2006) combined electrocardiography and respiratory activity recorded during the recall and experiential reliving of one or two potent emotional autobiographical episodes and a neutral episode. However, the use of it must be carefully integrated with other information gathering methods, as the information must be properly synchronized, which is not always an easy task. Besides, non-invasiveness of used signal detection devices must also be taken into account.

### Self-regulation

The use of biometric signals has allowed us to find people who control their own emotions while being tested. Self-control of emotions may be due to a conscious effort or be a learned skill. For instance, in the education of medical doctors, role-playing techniques are used in which students are educated to play a role and they learn how to control their emotions. It can lead to showing flat EDA activity.

A priori, it seems that people with self-control tend to look directly into the eyes. Moderated phasic activity is related to values below 50% of gaze eye fixations, which theoretically correspond to less empathic people. In order to detect if it is true affective empathy or just people trying to imitate a behavior, which would correspond to the cognitive empathy facet, more variables such as mimicry should be correlated. In fact, facial mimicry is related to muscles activity and there is a correspondence between the facial muscles and Ekman's six basic emotions: happiness, fear, anger, sadness, disgust, and surprise (Matzke et al., 2013). Flat responses on EDA values correspond with no mimicry in the 90% of cases.

Video cameras and software to establish facial movement have been helpful to detect mimicry. Emotiv EPOC EEG can also be used for this because it also includes two facial muscles sensors. In this sense, data redundancy is found to be convenient.

For our surprise, we discovered than twenty-one users controlled his/her EDA activity. Their EDA activity was normal before we told them that the experiment was to begin. Afterwards, it became flat or nearly zero as it can be visualized in Figure 3, where EDA decreases and decreases till drawing a very flat “U,” nearly zero. We have tried to find some profiles with some equal characteristics as we are going to see in the following paragraphs.

### Machine learning algorithm application

Machine learning algorithms have been previously applied to EEG signals to recognize emotions in human beings. For instance, they have shown that power spectrum across all frequency bands extracted from EEG signals perform well on distinguishing positive and negative emotional states (Wang et al., 2014). Different kinds of algorithms have been used, including SVMs (Liu et al., 2014), Neural Networks (Peng et al., 2005), or K-NN (Wang et al., 2011). Still, no references have been found by the authors in the literature dealing with self-regulation, emotions and machine learning. Therefore, it has been necessary to consider as many algorithms as possible to determine the validity of the proposed process, including preprocessing and synchronization.

## Conclusions and future work

This work presents a method to improve EEG based emotion recognition by combining it with synchronized biometric and eye tracking technologies in a non-invasive and low cost way. The method is aimed at combining these techniques, adequately synchronizing their data sources, in ICT environments. Gathered information has been used to detect different emotions the users experienced while watching a predefined set of stimuli based on the IAPS set of images and the MindReading corpus of videos. The equipment used to monitor users experience was Emotiv for EEG activity, Tobii Eye-Tracker and the Empatica wristband with sensors for Electro-Dermal Activity, Blood Volume Pulse, Acceleration, Heart Rate, and Temperature. This kind of equipment is relatively low cost and is the only one required to reproduce the study. After exposing users to stimuli and monitoring their reactions using the previous equipment, the gathered data has been synchronized and analyzed in the ways described in subsections Data Managing and Data Analysis Remarks using *ad-hoc* scripts.

We have been able to determine the basic emotions participants were experiencing by means of valence and arousal. Besides, proposed method has been able to detect empathy complex emotion. Obtained results show that it is possible to adequately combine different data sources to detect emotions with devices capable of being used in ICT environments. Besides, including different types of signals provides different ways to discard data when seemingly contradictory information is gathered.

Performed study has allowed us to gather some interesting conclusions.

- On the one hand, we have found evidence of EDA manipulation or self-control.
- An elevated percentage eye gaze fixations (>80%) could be interpreted as inquisitive rather empathic.
- Flat responses on EDA values correspond to users with no mimicry in a 90% of the cases. This suggests that their empathy is more developed in their cognitive facet that in their emotional one.

Furthermore, the machine learning study that has been performed has allowed determining that EEG alone is not a good indicator of self-regulation when processing affective stimuli. This supports the hypothesis that different biometric signal data sources should be considered together in order to determine self-regulation related to emotional stimuli.

As future work, it is foreseen to further improve the pre-test in order to detect deafness and personality traits such as shyness prior to the beginning of future studies. Apart from that, the use of machine learning techniques will allow us to predict an accurate valence degree. In this sense, EEG alone has proved to be a useful technique to indicate frontal alpha asymmetry and verify the pleasant/unpleasant dichotomy. Still, machine-learning algorithms should consider information of all data sources and data should be precisely preprocessed. Finally, a correlation with psychometric indicators as Empathy Quotient (EQ), Interpersonal Reactivity Index (IRI), Jefferson Scale of Physician Empathy (JSPE; Hojat et al., 2005), and NEO Five Factor Inventory (NEO-FFI) (Inchausti et al., 2015) is intended to be performed to fit personality profiles. Apart from its application to ICT environment, it would also be helpful for preventing diseases and mental disorders.

## Author contributions

JL explored different ways for emotion recognition, including sources of data from different signal measurement devices. He also helped defining the experimental protocol and providing ways to appropriately analyse gathered data. JV performed the device calibration and synchronization, data collection and facilitated the study. RGil coordinated the analysis of gathered information. She has found ways to expand it to medical environments. RGarcía participated in the definition of the protocol and explored ways to improve it. TG, IB, and JS recruited the medicine students that participated in the study and dealt with the required authorization by the ethics committee of the Arnau de Vilanova University Hospital. They also obtained the images and videos used in the study and supervised the experiments.

### Conflict of interest statement

The authors declare that the research was conducted in the absence of any commercial or financial relationships that could be construed as a potential conflict of interest.

## Footnotes

^1^http://www.choosemuse.com.
