# Corrigendum: Method for Improving EEG Based Emotion Recognition by Combining It with Synchronized Biometric and Eye Tracking Technologies in a Non-invasive and Low Cost Way

**DOI:** 10.3389/fncom.2016.00119

**Published:** 2016-11-29

**Authors:** Juan-Miguel López-Gil, Jordi Virgili-Gomá, Rosa Gil, Teresa Guilera, Iolanda Batalla, Jorge Soler-González, Roberto García

**Affiliations:** ^1^Department of Computer Languages and Systems, University of the Basque CountryVitoria-Gasteiz, Spain; ^2^Department of Computer Science and Industrial Engineering, Universitat de LleidaLleida, Spain; ^3^Psychiatry Service, Santa Maria University HospitalLleida, Spain; ^4^Biomedical Research Institute of LleidaLleida, Spain; ^5^Department of Medicine, Faculty of Medicine, Universitat de LleidaLleida, Spain; ^6^Institut Català de la Salut IDIAPBarcelona, Spain

**Keywords:** emotions, EEG, eye tracking, biometric information, empathy

Reason for Corrigendum:

Due to an oversight, there were errors in the list of authors, correspondence author, acknowledgments, and author's contributions. These things have been fixed in all respective sections, as agreed with the rest of the authors and the research topic editor. The authors would like to apologize for any inconvenience caused and this change does not affect the scientific conclusions of the article in any way. The original article has been updated.

## Author contributions

JL explored different ways for emotion recognition, including sources of data from different signal measurement devices. He also helped defining the experimental protocol and providing ways to appropriately analyse gathered data. JV performed the device calibration and synchronization, data collection and facilitated the study. RGil coordinated the analysis of gathered information. She has found ways to expand it to medical environments. RGarcía participated in the definition of the protocol and explored ways to improve it. TG, IB, and JS recruited the medicine students that participated in the study and dealt with the required authorization by the ethics committee of the Arnau de Vilanova University Hospital. They also obtained the images and videos used in the study and supervised the experiments.

### Conflict of interest statement

The authors declare that the research was conducted in the absence of any commercial or financial relationships that could be construed as a potential conflict of interest.

